# A Ligand-observed Mass Spectrometry Approach Integrated into the Fragment Based Lead Discovery Pipeline

**DOI:** 10.1038/srep08361

**Published:** 2015-02-10

**Authors:** Xin Chen, Shanshan Qin, Shuai Chen, Jinlong Li, Lixin Li, Zhongling Wang, Quan Wang, Jianping Lin, Cheng Yang, Wenqing Shui

**Affiliations:** 1 College of Life Sciences, Nankai University, Tianjin 300071, China; 2 High-throughput Molecular Drug Discovery Center, Tianjin Joint Academy of Biotechnology and Medicine, Tianjin 300457, China; 3 State Key Laboratory of Medicinal Chemical and Department of Pharmacy, Nankai University, Tianjin 300071, China; 4 Tianjin Institute of Industrial Biotechnology, Chinese Academy of Sciences, Tianjin 300308, China

## Abstract

In fragment-based lead discovery (FBLD), a cascade combining multiple orthogonal technologies is required for reliable detection and characterization of fragment binding to the target. Given the limitations of the mainstream screening techniques, we presented a ligand-observed mass spectrometry approach to expand the toolkits and increase the flexibility of building a FBLD pipeline especially for tough targets. In this study, this approach was integrated into a FBLD program targeting the HCV RNA polymerase NS5B. Our ligand-observed mass spectrometry analysis resulted in the discovery of 10 hits from a 384-member fragment library through two independent screens of complex cocktails and a follow-up validation assay. Moreover, this MS-based approach enabled quantitative measurement of weak binding affinities of fragments which was in general consistent with SPR analysis. Five out of the ten hits were then successfully translated to X-ray structures of fragment-bound complexes to lay a foundation for structure-based inhibitor design. With distinctive strengths in terms of high capacity and speed, minimal method development, easy sample preparation, low material consumption and quantitative capability, this MS-based assay is anticipated to be a valuable addition to the repertoire of current fragment screening techniques.

Over the past decade, fragment-based lead discovery (FBLD) has emerged as a paradigm-shifting strategy for the discovery of lead compounds for drug development, especially toward traditionally challenging yet therapeutically attractive targets^1^,^2^. In contrast to traditional high-throughput screens (HTS), FBLD involves the identification of low molecular weight “fragment” hits (<250–300 Da) bound to the target protein and their further elaboration into high affinity and high potency drug leads^3^,^4^. The increasing success of FBLD approaches is widely attributed to the higher ligand efficiency and improved chemical tractability of small-sized fragments compared with the larger and structurally more complex hits identified by high-throughput screening (HTS)^5^,^6^. Growing and linking fragment hit scan, therefore, explore greater chemical space, thus rending FBLD more effective in addressing targets intractable in conventional HTS campaign. The successful implementation of FBLD has been exemplified by the first FDA-approved fragment-based drug, Vemurafenib, for the treatment of metastatic melanoma, and a line of fragment-derived compounds in clinical trials^7^.

The weak interaction between fragments and protein targets (usually in the high micromolar to millimolar range) requires very sensitive methods for detection of bound fragments and characterization of their binding properties. A wide array of biophysical techniques have been employed in the screening stage of FBLD, notably differential screening fluorimetry (DSF)^8^, nuclear magnetic resonance (NMR)^9^,^10^, surface plasmon resonance (SPR)^11^, isothermal titration calorimetry (ITC)^12^,^13^ and X-ray crystallography^14^,^15^. Unfortunately, these current techniques are associated with one or another drawback such as high sample consumption, low throughput, immobilization of proteins, dynamic range limitations and occurrence of false positives or false negatives^16^. Therefore, an efficient fragment screening cascade has to combine several orthogonal technologies for reliable detection and characterization of fragment binding. A representative three-stage cascade established by Ciulli and his coworkers involves DSF for preliminary screening, NMR for hit validation, ITC and X-ray crystallography for binding characterization^8^,^17^. Given the aforementioned limitations of most current techniques, any additional approach with distinctive advantages is expected to expand the repertoire of available methods, increase the flexibility of building an integrated pipeline, and enhance the confidence in the fragment hit.

Mass spectrometry (MS)-based assays constitute an attractive addition to the arsenal of drug discovery techniques, with strengths lying in high sensitivity, selectivity, rapid and simultaneous measurement of multiple ligands^18^,^19^,^20^,^21^,^22^. Native MS analysis of the protein-ligand complexes allows for determination of both binding stoichiometry and dissociation constants (*K*_d_)^23^,^24^. An automated screen assay based on nanoeletrospray native MS has been demonstrated for rapid and sensitive detection of ligands with *K_d_* in the typical range of fragment binders^16^. However, several drawbacks of native MS analysis such as rigorous binding assay conditions (*e.g.*, buffer, pH, detergents), signal suppression, gas-phase dissociation, and non-specific binding largely hampers its applicability to diverse biological systems^25^,^26^,^27^,^28^. Instead of measuring the protein complex, ligand-observed MS detects ligands released from the protein complexes in a robust, sensitive and selective manner. The ligand-bound protein complexes can be purified by ultrafiltration^29^,^30^, size exclusion chromatography^31^, or affinity chromatography^32^,^33^ prior to LC-MS analysis. Although successfully implemented to screening combinatorial libraries and natural products for bioactive discovery^29^,^30^,^31^,^34^,^35^, the potential of ligand-observed MS in identification of low-affinity fragment hits has been rarely explored. A relevant example is the application of weak affinity chromatography to screening a fragment library targeting cyclin G-associated kinase, yet this approach required immobilization of the protein target and screening was executed in a relatively low throughput^32^. Furthermore, unlike the native MS characterization of protein complexes, the ligand-observed MS analysis is generally considered incapable of measuring dissociation constants of target-ligand pairs due to little correlation between MS responses and actual concentrations of bound ligands in solution. In fact, previous studies using this approach all ranked the relative order of binding affinity without *K*_d_ determination^29^,^30^,^36^,^37^.

In this study, we presented a ligand-observed MS approach for high-throughput screen of a 384-member fragment library as well as reliable estimation of binding affinity of each fragment hit. This approach was integrated into a FBLD program targeting the RNA polymerase of Hepatitis C virus (HCV), NS5B, which is a particularly attractive target for anti-HCV drug development^38^,^39^,^40^. SPR and X-ray crystallography have been implemented to identify fragment ligands which were then optimized to become a promising candidate for clinical evaluation^41^,^42^,^43^. The proposed discovery pipeline combining our MS-based approach and other biophysical techniques resulted in identification and validation of ten new fragments bound to two distinctive domains of NS5B, among which five were successfully translated to X-ray structures of fragment-bound complexes valuable for structure-based design of potent inhibitors.

## Results

### Ligand-observed MS screening and validation of the fragment library

**Figure 1:**
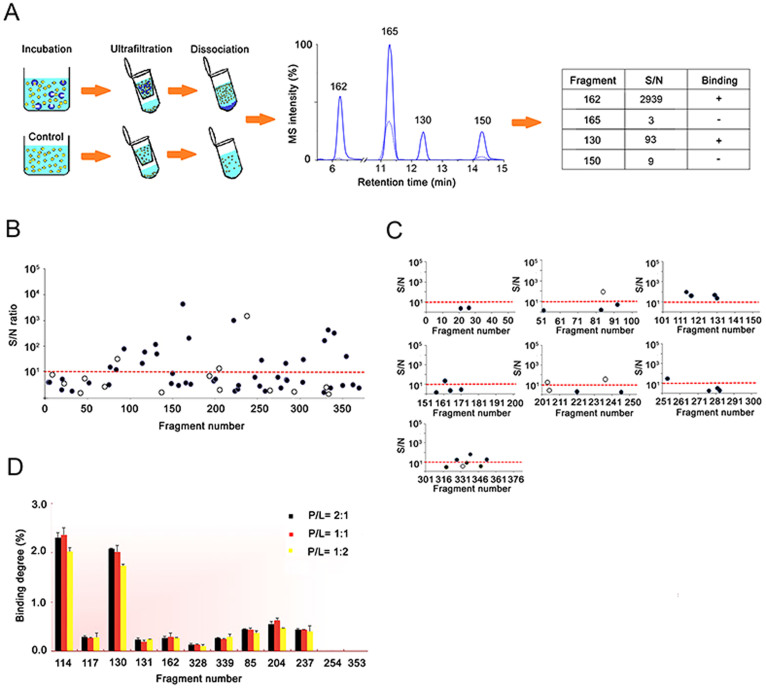
Fragment library screening based on the ligand-observed LC/MS approach. (A) Scheme of the MS-based fragment screening workflow. The protein is incubated with a mixture of the fragment library, ligand-bound complexes are purified by ultrafiltration and the dissociated ligands are identified and quantified by LC/MS. Positive fragment binding is indicated by its S/N ratio > 10. (B) Results of the primary screen of a 384-member fragment library in a single run. Solid and open circles designate data points acquired in the positive and negative modes of MS analysis respectively. Circles above the threshold line denote positive hits. S/N ratios are averages of experimental duplicates. (C) Results from the secondary screen of 7 subdivided fragment cocktails, each consisting of 50 or 86 members. A total of 12 hits were identified in this run. S/N ratios are averages of experimental duplicates. (D) Binding degrees of individual fragments measured from the 12-hit mixture at different protein/ligand ratios (P/L) while the protein concentration was held constant in incubation. Ten fragments were validated to bind to NS5B and their binding degrees indicate a relative order of binding affinity. Error bars denote s.d. from experimental duplicates.

A two-stage fragment screening strategy based on the ligand-observed MS analysis was devised that involved a primary screen of 384 fragment as a whole followed by a secondary independent screen of fragment cocktails of less complexity. In the primary screen, a mixture of all 384 fragments was incubated with NS5B protein before the fragment-bound complexes were separated from excessive free fragments using ultrafiltration. Notably, purification of protein complexes in solution by ultrafiltration eliminates the need of protein immobilization and thus preserves the native conformation of the target protein during its interaction with fragments. Fragments dissociated from the target using 90% methanol in deionized water were subjected to LC-MS analysis and those showing MS intensity substantially higher than the negative control (protein-free incubation) were considered positive hits (Figure 1A). Although in our previous assay development we justified an S/N ratio of 2 to be the threshold for positive binding^25^, here we raised the S/N threshold to 10 so as to retain the upmost strong binders given that low-affinity fragments are more inclined to nonspecific adsorption. Impressively, the high selectivity of high-resolution MS (HRMS) analysis enabled rapid screening of the 384-fragment mixture within 20-min analytical time, generating 20 preliminary hits with S/N above 10 (Figure 1B, full data sets in Table S1). It is noted that 20 compounds (5%) in this library are not amenable to our analysis, probably due to the limited sensitivity of the mass spectrometer used in this study or the intrinsically low ionization efficiency of these compounds. Nevertheless, these undetectable fragments were still included in the screening cocktail to maximize the complexity of multiple ligand interactions during incubation. To the best of our knowledge, such a high capacity in mixture screening has never been shown before for fragment libraries, and we anticipate the throughput can be easily boosted to screening >1000 fragments within half an hour using a state-of-the-art mass spectrometer.

To reduce possible false-positives from the primary screen, the library was divided to 7 cocktails among which six consist of 50 fragments and the last one of 84 fragments. Individual cocktails were incubated with NS5B protein prior to protein complex isolation and ligand-observed MS analysis, resulting in a total of 12 positive hits after screening the seven cocktails (Figure 1C). Importantly, all the hits acquired from the secondary screen fell into a subset of hits from the primary screen, suggesting sufficient reproducibility between the two independent screens (Table S1). Thus the confidence in hit identification was enhanced by selecting hits repetitively found from the two screens against cocktails of different diversity. It is noteworthy that the preliminary hits missed in the secondary screen might be due to either nonspecific binding or co-operative binding that only occurred when the entire library was incubated with the target. Apart from the 12 hits, 25 fragments with S/N < 10 from the secondary screen were regarded non-binders and discarded. The hit rate of 3.1% for the secondary screen is similar to typical hit rates observed in fragment-screening campaigns (2.4% ~ 3.2%) against diverse targets using the DSF technique^44^,^45^, indicating that our choice of the S/N cutoff and implementing a secondary screen resulted in a reasonable number of hits for downstream validation.

### Binding affinity measurement using our MS-based approach and validation by SPR

**Figure 2:**
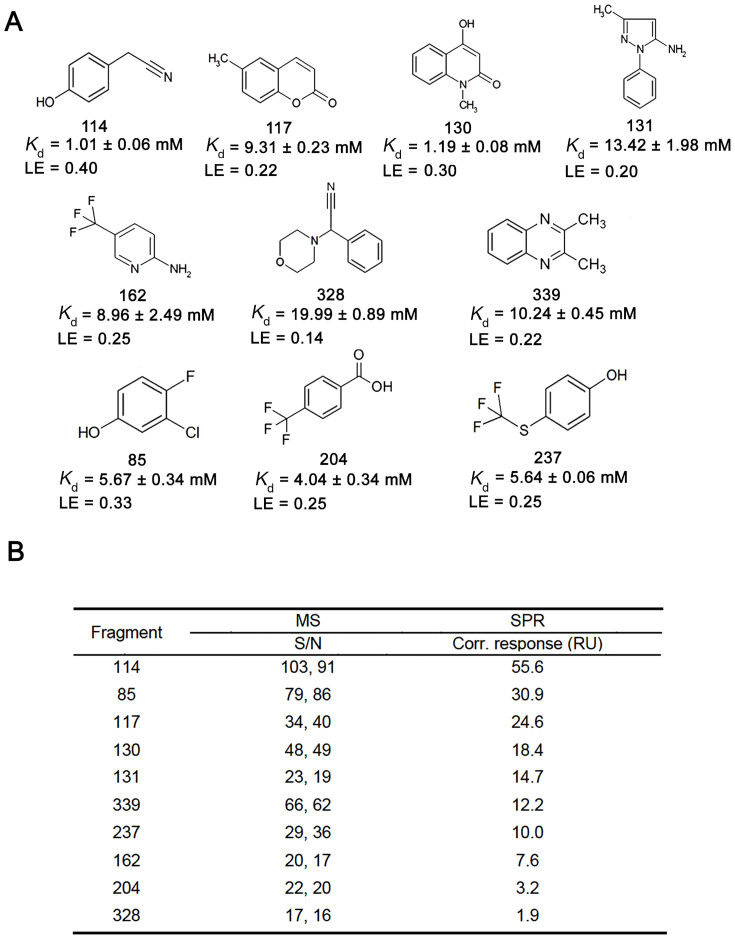
Quantitative assessment of binding affinity of fragment hits. (A) Chemical structures, estimated dissociation constants (*K*_d_) and ligand efficiency (LE) of the 10 hits. *K*_d_ calculation is based on ligand concentration measurement from the third-round validation assay (see Methods and Results for details). (B) S/N factors measured in the validation assay and SPR responses of the ten fragment hits. S/N factors from two independent experiments are shown. In SPR analysis, the corrected response above 5 RU indicates positive binding and the ranking order is a measure of relative binding affinity.

The twelve hits acquired from the secondary screen were then subjected to a third round of incubation and ligand-observed MS analysis for further validation and quantitative assessment of binding affinity. Analysis of this simplified mixture validated ten fragments binding of the NS5B target (Figure 1D and Table S1). Binding degree of each fragment represented by the fraction of fragment ligands over the total amount of fragments during incubation reflects the relative order of binding strengths. Keeping the protein concentration constant and increasing the protein:ligand mixing ratio (P/L) from 2:1, 1:1 to 1:2 did not significantly change the binding degrees of ten fragment hits (Figure 1D). This was within our expectation as theoretical calculation indicated that a fragment of low affinity around 1 mM would have a constant binding degree at 2.4% under the conditions of our assay. The measured binding degrees of fragments 114 and 130 were close to the theoretical value (Figure 1D). Structure wise, the 10 hits all contained 6-membered aromatic rings including fused 6-6 membered rings in 3 hits (Figure 2A). A prominent feature for another 3 hits was the presence of a trifluoromethyl group which is responsible for key interactions with active domains as discussed later. Physicochemical properties of the 10 fragment hits are provided in Table S2.

In order to determine the dissociation constant of each fragment ligand, we prepared calibration curves for the MS responses of ten fragments in a mixture (Figure S1) and measured the recovery rate of each fragment (Table S3), which were used to derive the actual concentrations of fragments bound to the target. *K*_d_ values were then calculated based on the measured concentrations of fragment ligands dissociated from the target (Table S3). It turned out that *K*_d_ of all fragment hits fell within the range of 1 mM to 20 mM at P/L of 1:1, suggesting very weak binding typically observed for fragments (Figure 2A). Ligand efficiency (LE) values were then derived from *K*_d_'s as a measure to justify which fragments to further optimize and elaborate in the FBLD pipeline^46^,^47^ (Figure 2A). To examine the accuracy of *K*_d_ measurement using this new approach, we first attempted to utilize ITC for binding affinity assessment. Unfortunately, ITC failed to produce binding isotherms probably due to very weak interactions or large flexibility of small ligand binding to the extensive allosteric sites of NS5B as reported previously^48^. SPR was then implemented for *K*_d_ determination for these fragment hits. Interestingly, eight out of the ten fragments were verified by SPR analysis to bind NS5B yet their binding affinity could not be precisely measured due to the weak signals in the sensograms (Figure S2). However, using the single point measurement method^49^, we were still able to estimate *K*_d_'s for these fragment-target interactions to be all within the millimolar range and rank the order of their binding affinity according to corrected responses (Figure 2B). Notably, the ranking of S/N factors measured by our MS-based approach was generally in accordance with that of SPR responses (Figure 2B). For fragment 328 showing SPR response below the typical threshold for positive binding, its affinity were estimated to be 20 mM by the MS-based analysis, suggesting the latter may be more sensitive in capturing very weak interactions. The overall consistency of affinity ranking and estimation between the two independent assays implied our new approach enabled reliable assessment of binding affinity, especially valuable for characterizing low-affinity fragments not amenable to conventional biophysical approaches.

### Validation and characterization of fragment binding by X-ray Crystallography

Similar to other nucleotide polymerases, the domain distribution of apo HCV NS5B polymerase bears an anatomical resemblance to a right hand with subdomains representing the fingers, thumb and palm^50^,^51^. Structural and mechanistic studies of NS5B have indicated the existence of multiple allosteric binding domains including palm I, II and III pockets and thumb I and II sites targeted by reported inhibitors^38^,^51^,^52^,^53^. To characterize the binding modes of the fragments studied, they were soaked in NS5B crystals to obtain the protein complex X-ray structures. We were able to resolve electron density corresponding to the soaked compound for five out of ten fragments (crystallographic data summary in Table S4).

**Figure 3:**
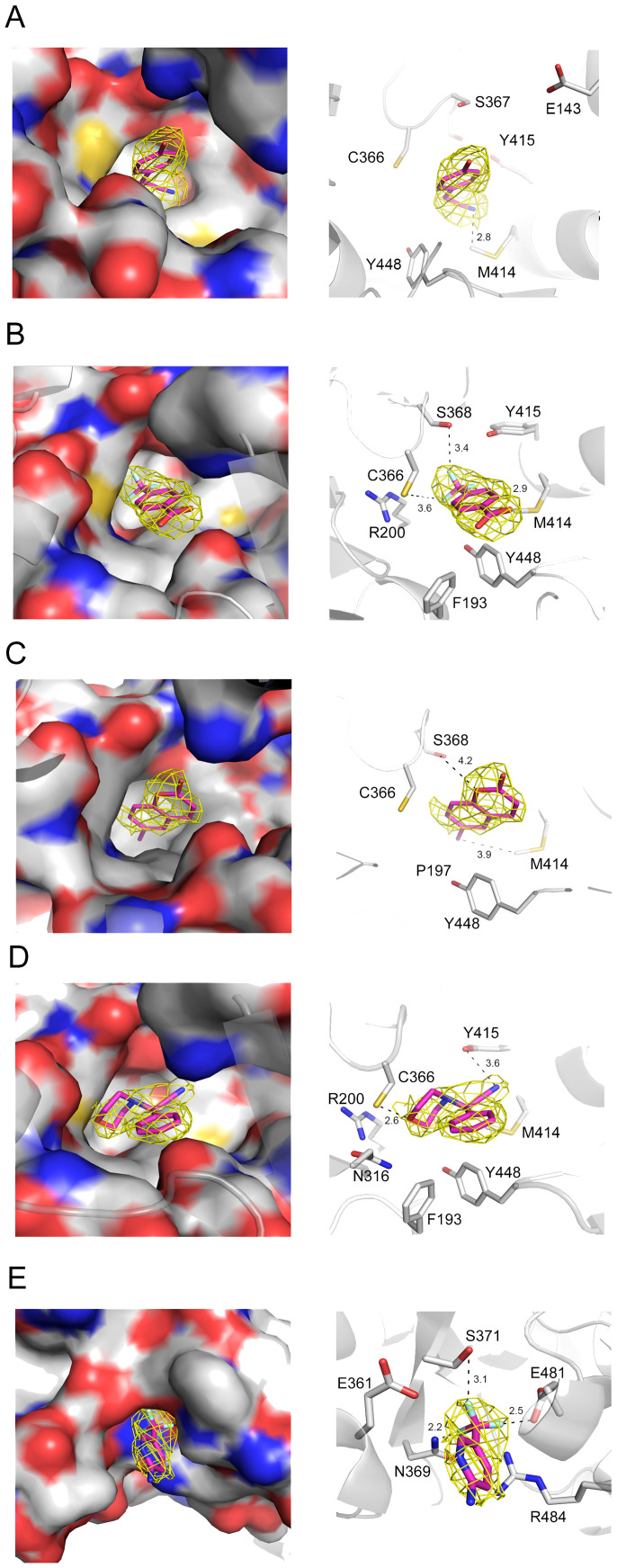
X-ray crystal structures of five fragments found to bind the allosteric site at the palm pocket (A, B, C, D) or at the thumb pocket (E). Crystal structures of bound fragments 114 (A), 204 (B), 117 (C), 328 (D) and 162 (E) are shown. The Fo–Fc omit electron density maps are shown as a yellow mesh contoured at 3σ around the fragments. Right panels in A, B, C, D and E depict the amino acids found to interact with the fragments, represented by dashed black lines. Carbon atoms are depicted by gray, oxygen by red, nitrogen by blue and sulfur by yellow colors.

Inspection of the crystal structures indicated that fragments 114, 204, 117, and 328 all bound at the palm I allosteric domain, while fragment 162 bound at the thumb region (Figure 3). Fragments 114 and 204 showed higher binding affinity than the others in the previous MS-based validation assay (Figure 2A). The X-ray structure of NS5B in complex with 114 revealed that its cyano group engages deep in a small hydrophobic pocket, where it forms hydrophobic interactions with Met414 and Tyr415. Moreover, the interaction could be enhanced by a π-π conjugate between its benzene ring and Tyr448 (Figure 3A). Fragment 204 engages deep in the palm I pocket through its trifluoromethyl moiety forming a hydrogen bond with Ser368 and van der Waals interaction with Cys366. Additionally, the hydrophobic interaction with Met414 as well as the π-π conjugate to Tyr448 may also contribute to the relatively high binding affinity of this fragment (Figure 3B). Fragment 117 binds at the pocket mainly through van der Waals interactions with Met414, Cys366 and Ser368 (Figure 3C), whereas fragment 328 forms van der Waals interactions with Tyr415 and Cys366 (Figure 3D). We assume these weak interactions may result in the lower binding strengths observed for the two fragments. Fragment 162 binds NS5B in a small cavity of the thumb pocket, with its trifluoromethyl moiety extending a key hydrogen bond with Asn369 as well as making hydrophobic interactions with Ser371 and Glu481. To our knowledge, the three residues constitute a new active domain in the thumb region never disclosed before.

### In-solution binding specificity evaluation

**Figure 4:**
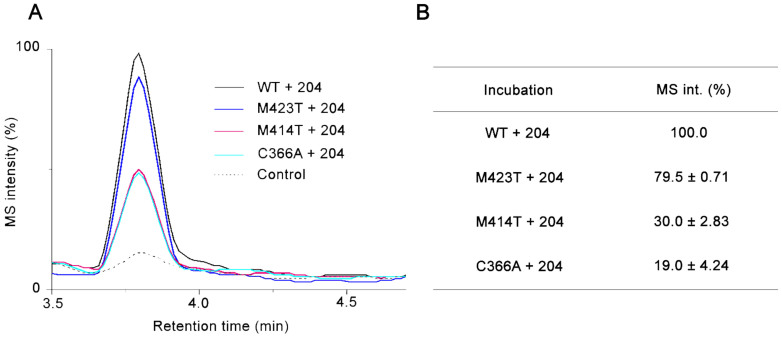
Determination of binding specificity of fragment 204 to NS5B in solution. (A) LC-MS chromatograms of fragment 204 detected from incubations containing the wild-type or specific mutants of NS5B, or from the protein-free control experiment. M414 and C366 are two residues observed to interact with the fragment in the crystal structure. M423 is speculated to have no interaction with the fragment. (B) Relative quantification for fragment 204 based on its MS intensity from (A) implies its interactions with M414T and C366A were significantly impaired whereas interaction with M423T was not, an evidence of binding specificity at these two sites. MS intensity percentages of fragment 204 from the mutant incubation relative to the wild-type incubation (defined as 100%) were averages from duplicate measurement and s.d. are shown accordingly.

To evaluate the binding specificity of fragment 204 to the palm domain of NS5B through interacting with certain residues indicated in the crystallographic data, we prepared two presumable binding-site mutants in the palm domain (C366A and M414T), and a control mutant in the thumb domain (M423T). Incubation of individual mutants with fragment 204 followed by ligand-observed MS analysis demonstrated the interactions between the fragment and the two binding-site mutants were substantially impaired by over 70% whereas the control mutant retained ~80% of the interaction with the fragment compared with the wild-type (Figure 4). Therefore, this experiment strongly implicated the finding by X-ray crystallography that fragment 204 binds to the palm domain of NS5B through interaction with Cys366 and Met414 was also valid in solution.

## Discussion

**Figure 5:**
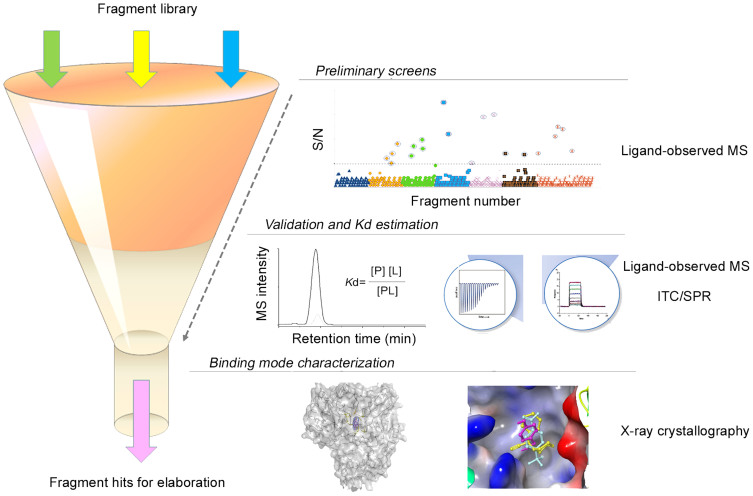
A fragment screening cascade incorporating the ligand-observed MS approach for efficient fragment hit identification. The MS approach shown in this study can play a major role at the stages of preliminary screens as well as hit validation and *K*_d_ estimation due to its supreme speed, selectivity, sensitivity and quantitative capability.

Our ligand-observed mass spectrometry analysis resulted in the discovery of 10 hits from a 384-member fragment library through two independent screens of complex mixtures and a follow-up validation assay. The secondary screen repetitively identified 12 out of the 20 hits from the primary screen, whereas 10 of them were confirmed in the final validation assay, indicating the advantage of hit rate enrichment through multi-round analysis. To expedite the discovery process, the secondary screen against less complex cocktails can be removed and preliminary hits will be directly validated by orthogonal techniques such as ITC, SPR or NMR. Importantly, this HRMS-based approach allows for not only high-throughput screens of a mixture of hundreds-to-thousands of fragments within half an hour but also quantitative assessment of binding affinity of individual ligands. The quantitative merit is particularly valuable for the evaluation of fragment ligands to NS5B for which binding properties cannot be assessed using the conventional ITC technique (our experience and mentioned in reference 48) or native mass spectrometry analysis of protein complexes due to technical limits (Figure S3). Furthermore, ranking and estimation of affinity for the 10 hits were in general consistent with the SPR data, and five of them were successfully soaked into the protein crystal to yield high-resolution diffraction data for structural elucidation of the protein-ligand interactions. According to the interaction modes of 3 fragments all observed to bind the allosteric site in the palm I pocket, we are currently designing new elaborated structures through fragment merging and growing for future evaluation and optimization. Taken together, our study presents a unique FBLD pipeline that integrates an efficient ligand-observed MS assay with other biophysical techniques such as SPR/ITC for binding validation and X-ray crystallography for binding mode characterization (Fig. 5). The MS-based assay shows distinctive strengths in terms of high capacity and speed, minimal method development, easy sample preparation, low material consumption and quantitative capability. It is a valued addition to the current fragment screening platform and will also find wide applications in ligand identification from natural product extracts or combinatorial libraries.

Several fragment hits first discovered by our approach shared structural similarity and binding characteristics with reported NS5B inhibitors. In this study, four fragment hits and the designed ligands all bound in the palm I pocket, where an overwhelming majority of NS5B inhibitors were also found to bind^52^,^53^ Specifically, Met414, Cys366 and Tyr448 in the palm domain of NS5B have been documented to be the “hot spots” for binding of high-potency inhibitors^38^,^54^ and they were also found to be the dominant sites for fragment binding in our study (Figure 3). Moreover, fragment 204 discovered in this study was a structural constituent of an effective inhibitor interacting with the palm region^52^,^53^. It is also noteworthy that another promising inhibitor targeting the palm I pocket was successfully designed by Talamas *et al*. based on a fragment hit from a library screen using both SPR and X-ray crystallography^41^. Therefore, the new fragment ligands as well as the elaborated molecules provided in this study would be anticipated to bolster efforts to optimize the current inhibitors and to develop other chemical series for combating HCV infection.

## Methods

### Protein expression and purification

NS5B with a C-terminal 10-His tag was expressed in *E. coli.* Site-directed mutagenesis was performed using the Quick Site-directed mutagenesis kit according to the manufacturer's instructions (Stratagene, China). All constructs were verified by DNA sequencing. The wild-type and mutant proteins (M414T, M423T and C366A) were first purified using a Ni-NTA column(GE Healthcare), followed by anionexchange chromatography on a Resource S column (GE Healthcare) and eluted with a solution of 20 mM 2-(N-morpholino) ethanesulfonic acid (MES) (pH 6.5), 1M NaCl, and 10% glycerol. Fractions were assayed by SDS-PAGE, and the purified NS5B was concentrated to 4–6 mg/mL and exchanged to a buffer of 20 mM MES (pH 6.5), 300 mM NaCl, and 10% glycerol before storage at −80°C.

### Ultrafiltration and ligand-observed LC/MS assay

In the primary screening, a mixture of 384 fragments (each at a final concentration of 25 μM) and NS5B at 50 μM was prepared in the binding buffer of 20 mM MES (pH 6.5), 300 mM NaCl and 10% glycerol, and incubated for 40 min at 25°C. All fragments of the library were either purchased from Sigma or synthesized in our laboratory, and their structural diversity was summarized in Figure S4. The mixture was then filtered through an ultrafiltration membrane (50 KD MW cut-off) by centrifugation at 13000 g for 10 min at °C. The protein complexes bound with different fragments were washed twice with 10 mM ammonium acetate (pH 6.5) followed by centrifugation to remove the unbound fragments. The resulting solution of the complexes was transferred to a new centrifugal tube, and the ligands were dissociated from NS5B with 90% methanol in deionized water. The released ligands were then separated from the denatured protein by centrifugation at 13000 g for 10 min at 20°C. The supernatant containing the ligands was evaporated by a speed vacuum, reconstituted in 80% methanol, and analyzed by LC/MS. The protein-free control was prepared by using the binding buffer substitute for NS5B during incubation and it underwent the same process as the NS5B incubated sample. In the secondary screen, 384 fragments were divided into 7 cocktails, each consisting of 50 fragments except for the seventh cocktail of 84 fragments. Each cocktail was then incubated with NS5B in the binding buffer at the same final concentration as in the primary screen. In the third assay for binding validation and *K*_d_ estimation, 12 fragment hits resulting from the secondary screen were incubated with NS5B at three different mixing ratios (P/L = 2:1, 1:1, 1:2), with a constant protein concentration at 25 μM and varying fragment concentrations. Ultrafiltration and complex dissociation were performed as described earlier to obtain fragment ligands. Samples were all prepared in duplicate and analyzed separately.

Aliquots of reconstituted ultrafiltrates were analyzed on a Waters Synapt G1 high-dfinition mass spectrometer (Milford, MA) equipped with a Waters Acquity UPLC system. UPLC separation was carried out on a Waters Acquity BEH C18 column (2.1 mm × 50 mm, 1.7 μm) at a flow rate of 200 μL/min, with the mobile phases of water/0.1% formic acid (A) and acetonitrile/0.1% formic acid (B). In the primary screen, the LC gradient was as follows: 0–15 min, 2–50% B; 15–17 min, 50%–90% B; 17–20 min, 90% B. In the secondary screen and the validation assay, the first fraction of gradient (2–50% B) was shortened to 6 min. The regular ESI ion source operated in both positive- and negative-ion modes. Mass spectra were acquired within a mass range from 100 to 800 m/z, with capillary voltage 3.0 kV in the positive mode and 2.6 kV in the negative mode, sample cone voltage 54 V (positive mode) and 45 V (negative mode), extraction cone voltage 4.7 V (positive mode) and 4.0 V (negative mode), desolvation temperature 350°C, source temperature 100°C, and desolvation gas flow 500 L/h. External calibration with a solution of sodium formate achieved mass accuracy within 10 ppm. LC/MS chromatogram extraction and peak area calculation for specific fragments were performed by use of MassLynx software (v4.1, Waters) based on accurate mass measurement of the compound with a tolerance of 0.01 Da. For display of multiple extracted ion chromatograms, the raw LC/MS data were exported into Origin 75 (Original Lab) for chromatogram reconstruction.

### *K*_d_ estimation

First, the concentration of a specific fragment in the mixture bound to NS5B protein ([L]_b_) was calculated using equation 1:
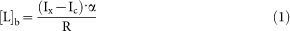
where I_x_ and I_c_ are the peak areas of the LC-MS chromatograms for the fragment released from the NS5B complex and the control sample respectively (both normalized with an internal standard), α is the calibration factor derived from the calibration curve of the fragment used for calculating concentration of dissociated fragments^55^, and R is the recovery rate of the fragment used to normalize its non-specific binding to the ultrafiltration membrane^56^. The calibration curves and recovery rates of individual fragments are provided in Figure S1 and Table S3.

Considering four allosteric binding sites available in NS5B and weak binding affinity of fragments to NS5B, we used the following equation 2 to estimate *K*_d_ for each fragment ligand when competition among the ligands and multiple ligand binding to the same site were ignored^58^. Binding stoichiometry of each fragment was assumed to be 1:1 as documented for most fragment ligands of diverse proteins including NS5B^8^,^17^,^41^,^57^.
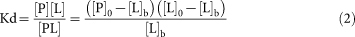
Where [P]_0_ and [L]_0_ are the initial concentrations of the protein and the ligand, [L]_b_ is derived from equation 1. Based on *K*_d_ estimation, we evaluated the ligand efficiency of each fragment hit using equation 3^59^.
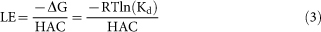
where HAC is the number of heavy atoms in the ligand.

### SPR analysis

The SPR experiment was performed on a Biacore T200 optical biosensor (Biacore Life Sciences, GE Healthcare). Series S sensor chips CM5, NHS, EDC, ethanolamine HCl as well as sampling vials and caps were all obtained from Biacore. A solution of 1 × PBS with 5% DMSO was used as running buffer. NS5B protein at 50 μg/ml prepared in buffer of 10 mM sodium acetate (pH 5.5) was injected for immobilization which reached a level of 8000 RU. During each binding cycle, the fragment solution was injected for 1 min at a flow rate of 30 μL/min and dissociation was monitored for 300 s. Data were collected with the biosensor instrument thermo stated to 25°C. Raw data collected on an SPR biosensor were further processed to remove systematic artifacts stemming from nonspecific binding, signal drift, and bulk refractive index changes. Solvent correction and molecular weight adjustment were also applied in this study. All data processing and analysis was performed using the Biacore T200 Evaluation Software.

### Protein crystallization and X-ray crystallography

The final optimized NS5B crystals were grown by the hanging drop method using a well solution of 30 mg/mL protein, 20% (w/v) PEG-4000, 10% (v/v) glycerol, 5 mM DTT and 50 mM MES (pH 5.5) and diffracted to 2 Å. Fragments were soaked onto the NS5B crystals at a final concentration of 5 mM (2.38% DMSO) for 2–3 days. Diffraction data were collected at Rigaku MicorMax 007HF X-ray diffraction instrument with an R-AXIS HTC detector (Rigaku), and processed using CCP4^60^. The NS5B structure was solved by molecular replacement using NS5B (1QUV) as the search model. The fragment-bound NS5B complex structures were determined by rigid-body refinement using the apo NS5B structure as a starting model^61^. Structure improvement proceeded with multiple rounds of manual model building in Coot, followed by refinement using CCP4. Fragment ligands were fitted into clear Fo-Fc electron density at the late stage of refinement. Images were rendered from PyMOL. Table S4 summarizes the X-ray crystallography data collection and refinement statistics.

## Author Contributions

X.C. and S.Q. planned and executed the experiments for protein purification, ligand-observed MS analysis, ITC and SPR, prepared figures and drafted the manuscript. S.C. planned and performed the experiment for X-ray crystallography and prepared figures. J.L. helped with data analysis. L.L. assisted the LC-MS analysis. Z.W. and Q.W. contributed important reagents. J.P. and C.Y. provided intellectual contributions and input to the manuscript. W.S. conceived of the study, directed the research and wrote the manuscript.

## Supplementary Material

Supplementary Information SI materials
