# Supplementary material for: A Ligand-observed Mass Spectrometry Approach Integrated into the Fragment Based Lead Discovery Pipeline
Source: Sci Rep. 2015 Feb 10;5:8361. doi: 10.1038/srep08361 (PMC4322365; doi:10.1038/srep08361)
Supplement: Supplementary Information — SI materials [file srep08361-s1.pdf]

Supporting information for the manuscript

A Ligand-observed Mass Spectrometry Approach Integrated into the Fragment Based Lead Discovery Pipeline

Xin Chen[1,2,#] , Shanshan Qin[1,2, #], Shuai Chen[1,2, #], Jinlong Li[1,3], Lixin Li[2], Zhongling Wang[1,2], Quan Wang[2], Jianping Lin[2,3], Cheng Yang[2,3]*, Wenqing Shui[1,4]*

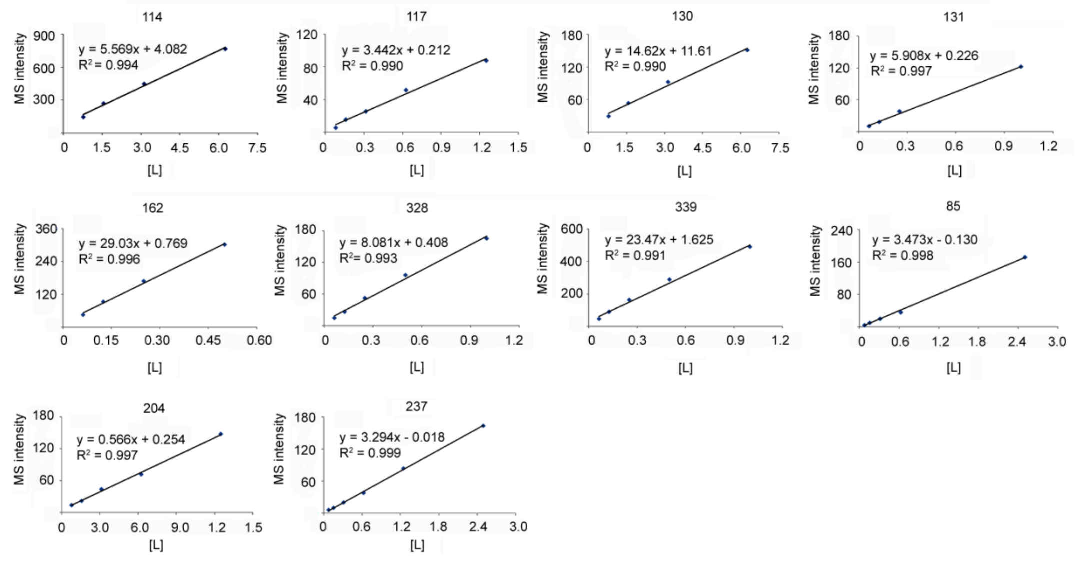

Figure S1. Calibration curves of the 10 fragment hits in a mixture. MS intensity of each fragment is plotted against its concentration in the mixture ([L]). Fragment numbers are shown above the plots. Each data point was from replicate measurements.

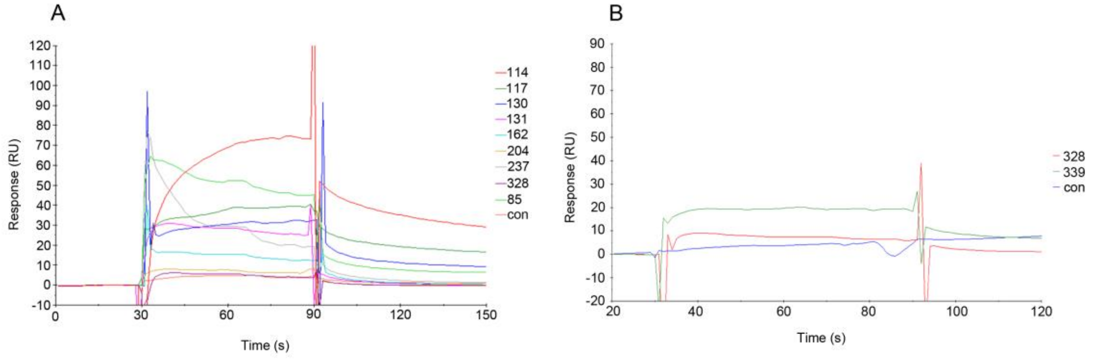

Figure S2. SPR analysis for fragment binding validation. Measured SPR sensorgrams of 9 fragment hits (A) and the tenth fragment 339 (B) interacting with NS5B. Fragments 204 and 328 in (A) are considered no binding from the SPR data (response <5 RU). The corrected SPR responses for all fragment hits are listed in Figure 2B.

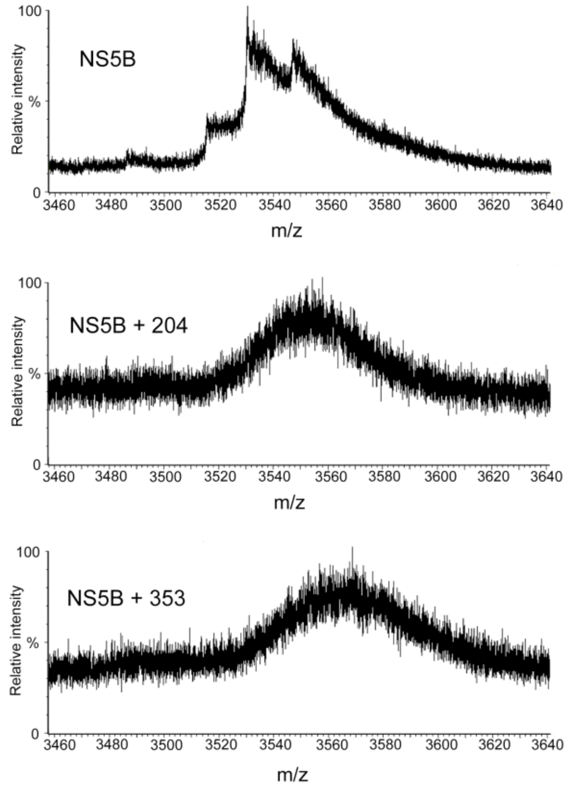

Figure S3. Representative MS spectra of apo-NS5B (top), NS5B incubated with fragment 204 (middle), or fragment 353 (bottom). *NS5B protein was buffer-exchanged to 50 mM ammonium acetate prior to the native protein analysis. The experiment was performed according to the previously described method from reference 25. The native protein MS analysis failed to detect ligand binding to NS5B due to very low resolution of the spectra.*

Figure S4. Structural diversity of the fragment library used in this study

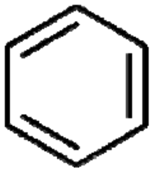	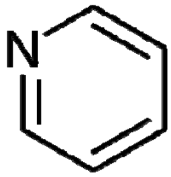	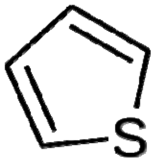
N = 96	N =30	N = 16
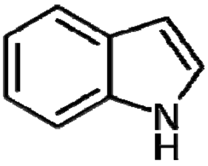	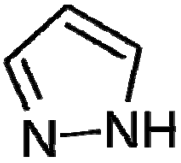	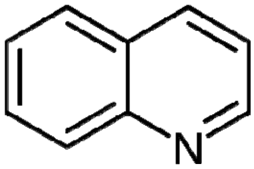
N = 11	N = 8	N = 8
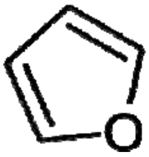	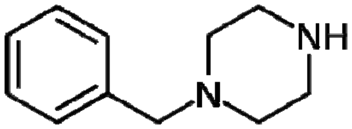	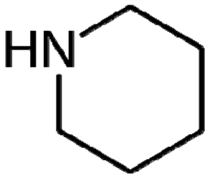
N = 7	N = 6	N = 5
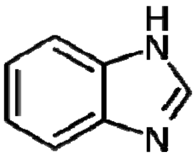	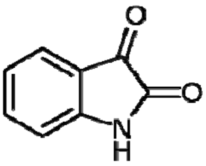	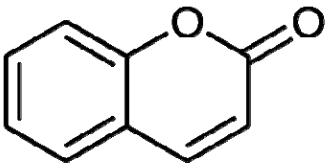
N = 5	N = 5	N = 5
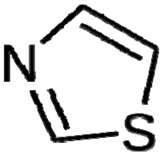	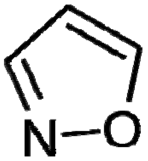	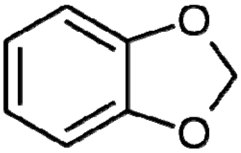
N = 4	N = 4	N = 4
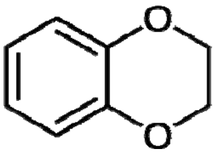	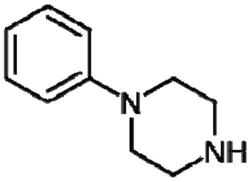	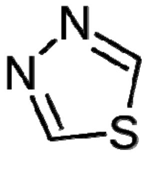
N = 4	N = 4	N = 3
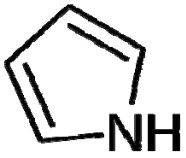	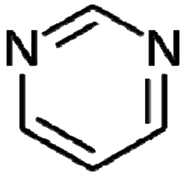	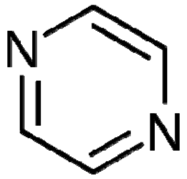
N = 3	N = 3	N = 3

Figure S4. Structural diversity of the fragment library used in this study (cont.)

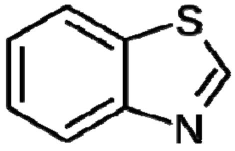	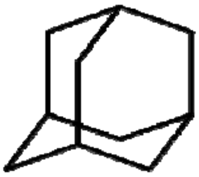	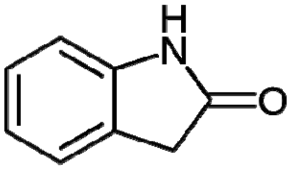
N = 3	N = 3	N = 3
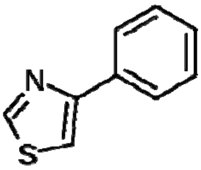	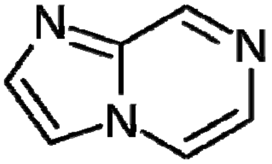	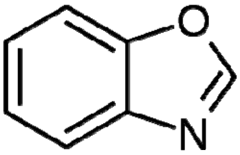
N = 3	N = 2	N = 2
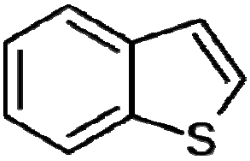	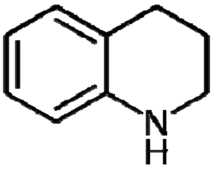	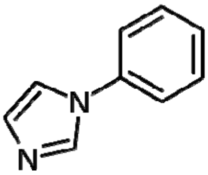
N = 2	N = 2	N = 2
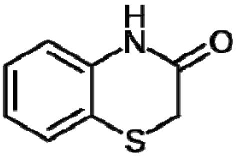	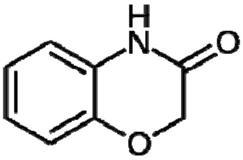	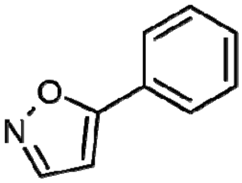
N = 2	N = 2	N = 2
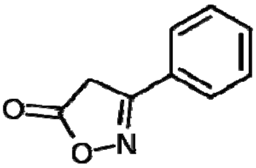	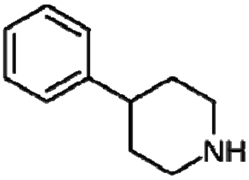	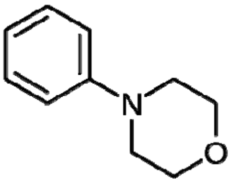
N = 2	N = 2	N = 2
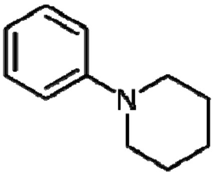	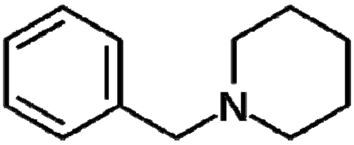	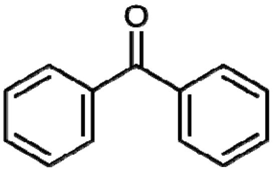
N = 2	N = 2	N = 2
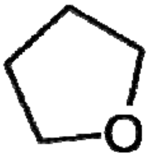	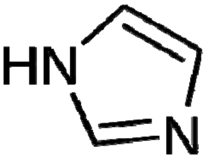	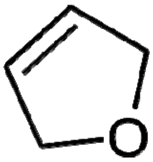
N = 1	N = 1	N = 1

Figure S4. Structural diversity of the fragment library used in this study (cont.)

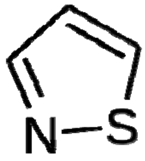	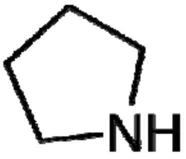	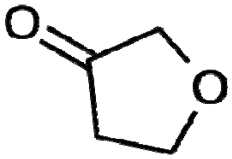
N = 1	N = 1	N = 1
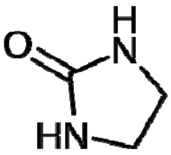	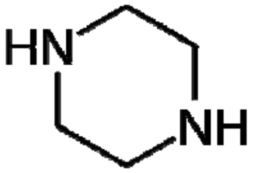	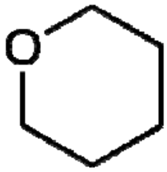
N = 1	N = 1	N = 1
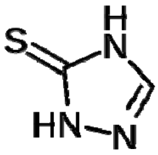	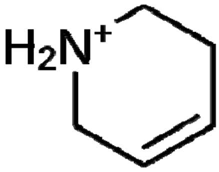	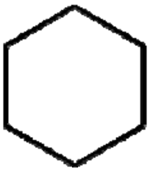
N = 1	N = 1	N = 1
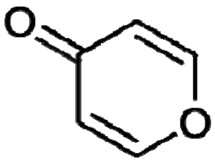	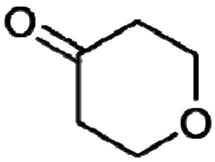	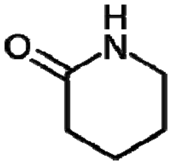
N = 1	N = 1	N = 1
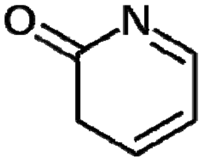	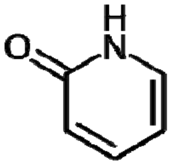	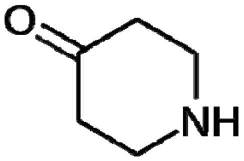
N = 1	N = 1	N = 1
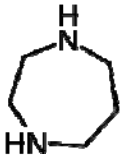	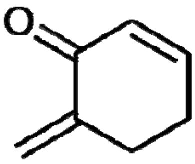	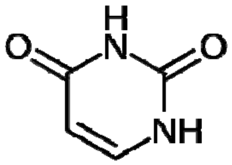
N = 1	N = 1	N = 1
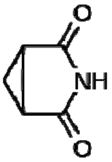	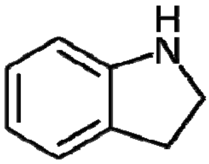	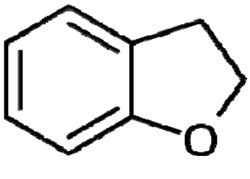
N = 1	N = 1	N = 1

Figure S4. Structural diversity of the fragment library used in this study (cont.)

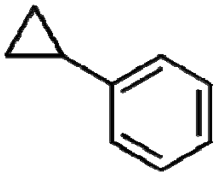	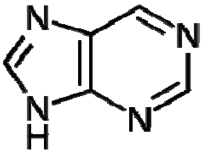	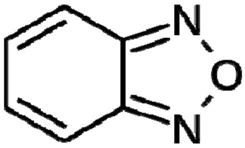
N = 1	N = 1	N = 1
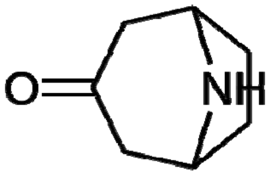	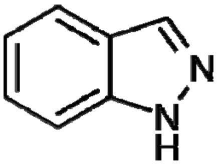	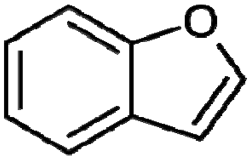
N = 1	N = 1	N = 1
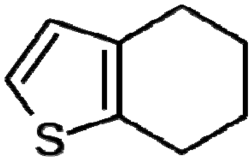	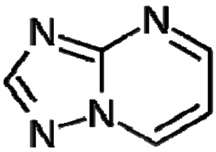	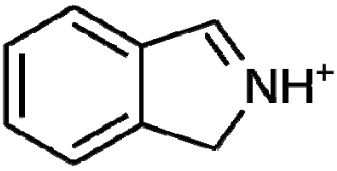
N = 1	N = 1	N = 1
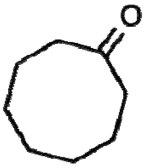	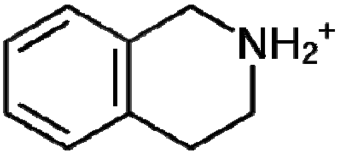	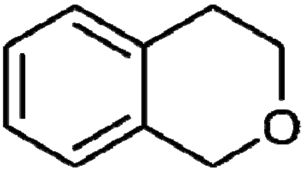
N = 1	N = 1	N = 1
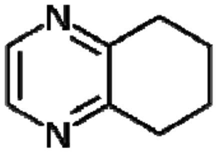	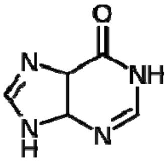	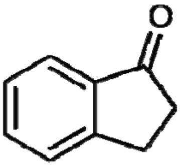
N = 1	N = 1	N = 1
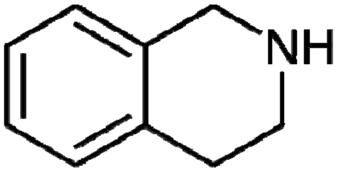	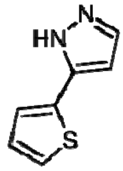	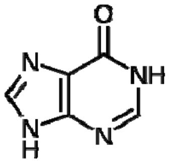
N = 1	N = 1	N = 1
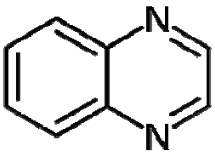	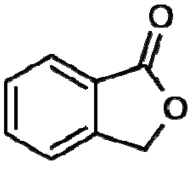	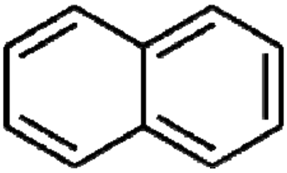
N = 1	N = 1	N = 1

Figure S4. Structural diversity of the fragment library used in this study (cont.)

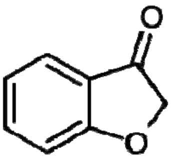	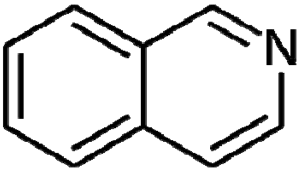	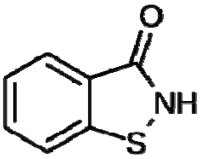
N = 1	N = 1	N = 1
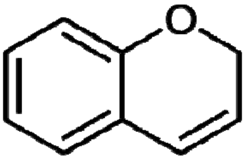	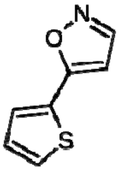	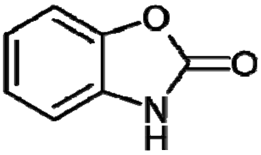
N = 1	N = 1	N = 1
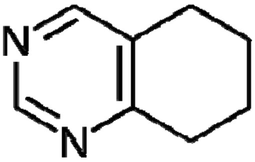	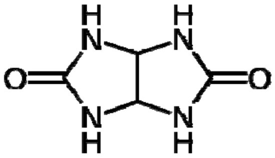	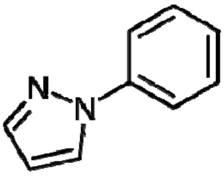
N = 1	N = 1	N = 1
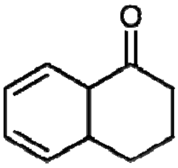	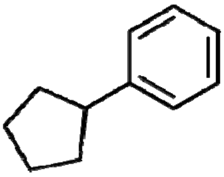	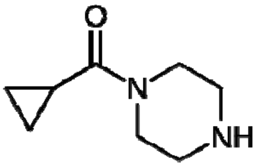
N = 1	N = 1	N = 1
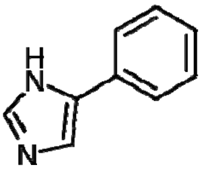	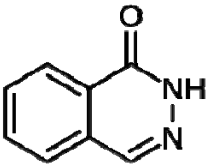	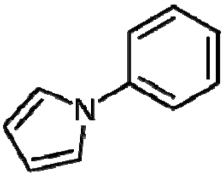
N = 1	N = 1	N = 1
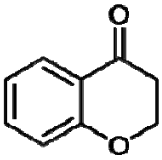	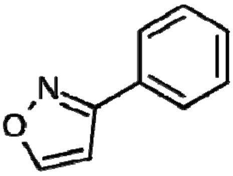	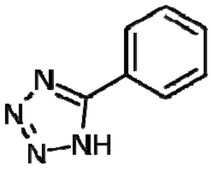
N = 1	N = 1	N = 1
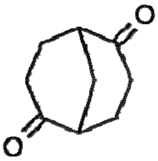	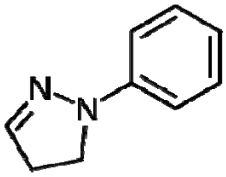	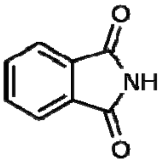
N = 1	N = 1	N = 1

Figure S4. Structural diversity of the fragment library used in this study (cont.)

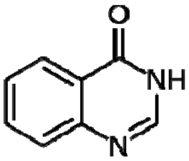	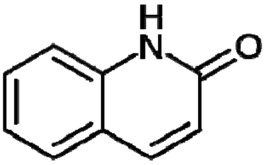	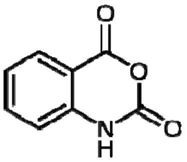
N = 1	N = 1	N = 1
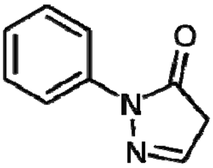	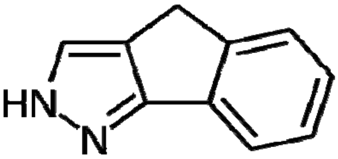	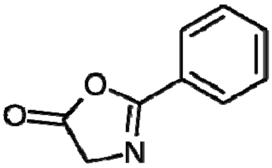
N = 1	N = 1	N = 1
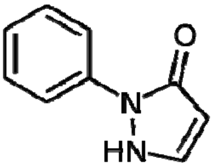	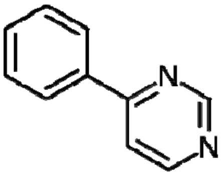	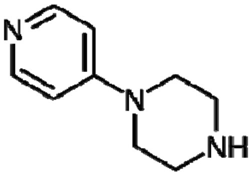
N = 1	N = 1	N = 1
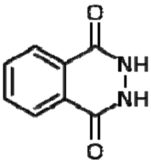	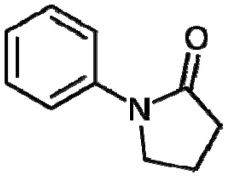	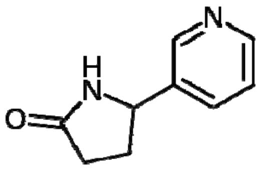
N = 1	N = 1	N = 1
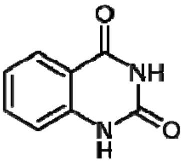	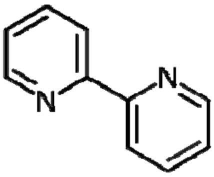	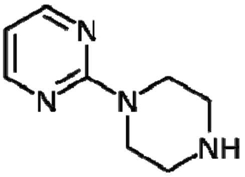
N = 1	N = 1	N = 1
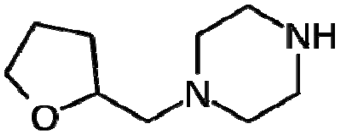	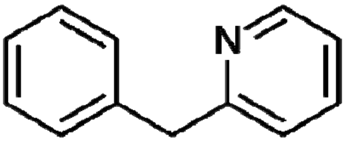	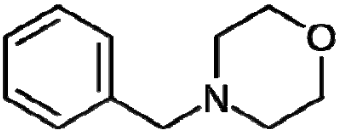
N = 1	N = 1	N = 1
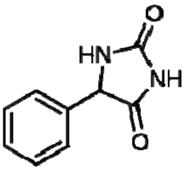	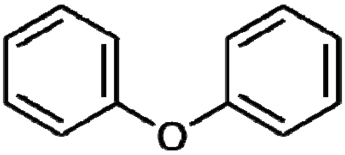	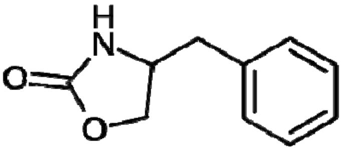
N = 1	N = 1	N = 1

Figure S4. Structural diversity of the fragment library used in this study (cont.)

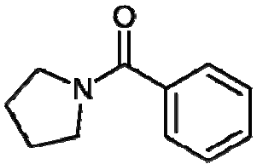	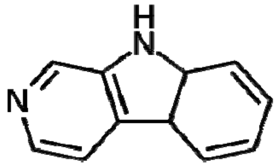	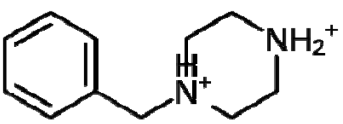
N = 1	N = 1	N = 1
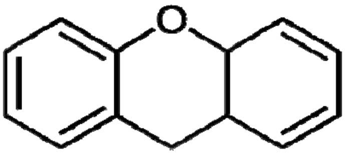	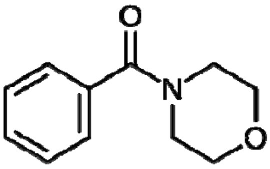	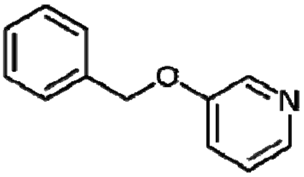
N = 1	N = 1	N = 1
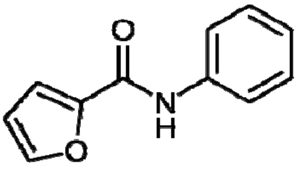	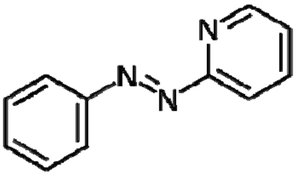	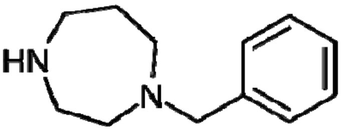
N = 1	N = 1	N = 1
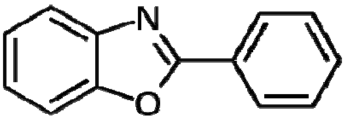	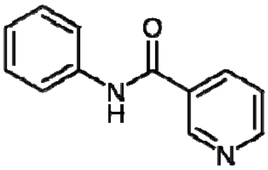	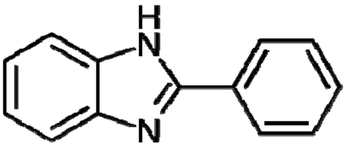
N = 1	N = 1	N = 1
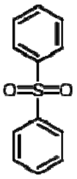	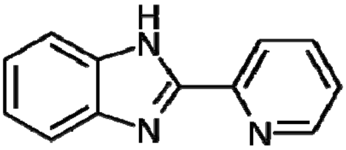	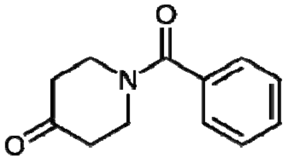
N = 1	N = 1	N = 1
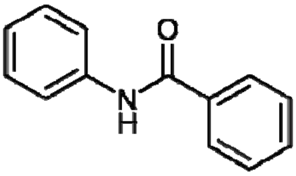	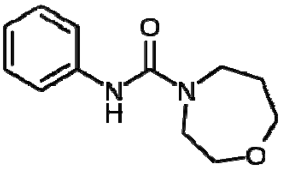	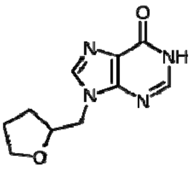
N = 1	N = 1	N = 1
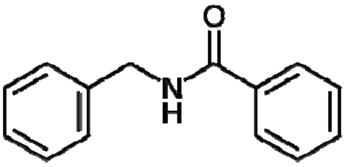	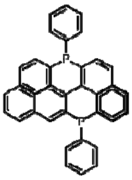	
N = 1	N = 1	

Table S1. S/N ratios of the 20 preliminary hits determined in the primary, secondary screens and the validation assay. *S/N > 10 indicates positive hits in each run.*

Fragment No.	S/N in the 1st screen	S/N in the 2nd screen	S/N in the validation assay
77	15.5/12.7	ND	-
84	10.4 /12.2	1.6/1.2	-
85	27.9/29.7	79.0/86.0	60.0/53.0
93	64.2/70.4	5.3/4.6	-
114	17.9/20.9	103.0/91.0	983.0/1198.0
117	55.6/46.8	34.3/39.6	111.0/122.0
130	93.1/102.3	48.4/49.4	378.0/376.0
131	45.7/39.3	23.1/19.4	56.0/46.0
162	2714/3280	19.7/17.3	466.0/513.0
169	157.6/179.4	ND	-
204	13.4/11.6	22.4/19.8	21.0/23.0
221	789.5/751.1	1.3/1.8	-
237	1027/1179	29.0/36.0	212.0/196.0
254	23.7/26.9	32.0/25.0	ND
282	21.4/17.8	2.7/1.9	-
303	25.2/27.8	ND	-
328	124.6/138.4	17.4/16.3	170.0/155.0
332	361.1/319.3	ND	-
339	241.4/259.8	66.2/62.4	172.0/223.0
353	32.5/35.9	16.0/13.0	ND

Notes:
1) Two individual S/N factors from experimental replicates were shown and separated by a slash;
2) ND: not detected by MS analysis
3) “-“ indicates the fragment was not included in the mixture for the validation assay.

Table S2. Physicochemical properties of the 10 fragment hits identified in this study

Physicochemical properties	Average	SD	Rule of three
Molecular weight, Da	169.5	21.8	<300
Polar surface area, Å2	37.4	11.3	≤60
Number of rotatable bonds	1.30	1.06	≤3
Hydrogen bond acceptor	2.22	1.36	≤3
Hydrogen bond donor	0.90	0.74	≤3
LogP	1.88	0.80	≤3

Table S3. Recovery rates and estimated K_d's of individual fragment hits from the MS-based validation assay

Fragment number	Recovery rate[a] (%)	K_d (mM)[b]		
		P/L= 2:1	P/L=1:1	P/L= 1:2
114	76.2	1.13±0.25	1.01±0.06	1.20 ±0.00
117	57.8	8.96±0.84	9.31±0.23	9.18±2.75
130	72.9	0.95±0.08	1.19±0.08	1.37±0.04
131	77.1	12.66±2.03	13.42±1.98	7.29±0.19
162	60.6	10.23±1.30	8.96±2.49	9.53±0.26
328	57.4	23.49±2.65	19.99±0.89	26.36±7.92
339	94.4	11.21±1.45	10.24±0.45	8.95±2.03
85	75.6	6.20±0.51	5.67±0.34	6.80±0.99
204	64.4	3.78±0.35	4.04±0.34	5.27±0.00
237	86.7	5.19±0.46	5.64±0.06	6.45±1.67

[a]The recovery rates were calculated by comparing the MS intensity of the fragment before and after ultrafiltration to reflect the extent of nonspecific binding to the filter membrane during ultrafiltration.
[b]K_d was calculated based on the recovery rate and the measured concentration of ligands released from the protein complex.

Table S4. Crystallographic data collection and refinement statistics for the five fragment-bound crystal structures obtained in this study

	fragment 114	fragment 117	fragment162	fragment 204	fragment 328
**Data Collection**					
Protein Data					
PDB ID code	4TXS	4TY8	4TY9	4TYA	4TYB
X-Ray Source	HighFluxHomeLab™ (Rigaku)	HighFluxHomeLab™ (Rigaku)	HighFluxHomeLab™ (Rigaku)	HighFluxHomeLab™ (Rigaku)	HighFluxHomeLab™ (Rigaku)
Space Group	$P2_12_12_1$	$P2_12_12_1$	$P2_12_12_1$	$P2_12_12_1$	$P2_12_12_1$
Cell Parameters (α=β=γ=90°)					
a (Å)	102.18	101.94	101.77	102.18	102.45
b (Å)	102.08	102.07	101.64	102.16	102.35
c (Å)	251.87	251.93	251.58	251.98	251.88
Resolution range,	50.00-2.78	50.00-2.78	50.00-2.78	50.00-2.94	50.00-2.94
Å (outer shell)	2.83-2.78	2.83-2.78	2.83-2.78	2.99-2.94	2.99-2.94
No. of unique reflections	66889	66811	66538	56755	57886
Multiplicity	6.2	4	6.5	4.5	4.9
R_{merge} % (outer shell)	6.6 (13.5)	12.1 (31.7)	11.9 (41.9)	10.8 (21.6)	10.6 (24.2)
Average I/σ (outer shell)	25.8 (8.8)	12.5 (2.9)	12.5 (2.6)	16.9 (4.5)	14.8 (3.9)
Completeness % (outer shell)	95.0 (85.1)	92.2 (88.8)	99.9 (100)	99.0 (91.6)	99.2 (98.1)
Mosaicity, °	0.564	0.503	0.527	0.634	0.514
**Refinement**					
Resolution range,	45.69-2.78	49.96-2.78	42.76-2.78	47.34-2.94	45.81-2.94
R_{cryst}, %*	21.96	20.73	20.73	20.09	20.52
R_{free},%†	29.36	26.80	27.48	27.03	27.26
Number of reflections					
Working set	63926	61826	66506	56172	57358
Test set	60467	58493	62833	53023	54197
Ligands bound to active site					
Ligand	4	2	3	4	4

*$R_{cryst} = \Sigma||F_{obs}|-|F_{calc}|| / \Sigma|F_{obs}|$, and F_{obs} and F_{calc} are observed and calculated structure factor amplitudes.
†R_{free} as for R_{cryst} using a random subset of the data excluded from the refinement.
